# Update on Meniscal Injury Treatment

**DOI:** 10.1055/s-0044-1787784

**Published:** 2025-11-21

**Authors:** Rafael Erthal de Paula, Bernardo Crespo Alves, Alan de Paula Mozella

**Affiliations:** 1 Knee Surgery Center, Instituto Nacional de Traumatologia e Ortopedia (INTO), Rio de Janeiro, RJ, Brazil

**Keywords:** meniscus, arthroscopy, knee injuries, knee, surgical procedures, operative, menisco, artroscopia, traumatismos do joelho, joelho, procedimentos cirúrgicos operatórios

## Abstract

Throughout the last few decades, the evolution of the surgical treatment of meniscal injuries has been remarkable, especially since a deeper understanding of the meniscus functions has come to light. This evolution resulted in a trend towards more conservative surgical approaches, focusing on preserving the meniscal anatomy as close as possible to the native status. Such a trend has led to a preference for meniscal repair procedures, which represents a significant change from previous practices. This transformation reflects the ongoing commitment to optimize the clinical and functional outcomes for patients with meniscal injuries.

## Resumo

Ao longo das últimas décadas, a evolução do tratamento cirúrgico das lesões meniscais tem sido notável, especialmente à medida que um entendimento mais profundo das funções desempenhadas pelo menisco vem à tona. Nesse contexto, observa-se uma tendência a realizar abordagens cirúrgicas mais conservadoras, centradas na preservação da anatomia meniscal da forma mais próxima possível ao estado nativo. Isso se traduz em uma preferência por procedimentos de reparo meniscal, o que representa uma mudança significativa em relação às práticas anteriores. Essa transformação reflete o compromisso contínuo de otimizar os resultados clínicos e funcionais para os pacientes que enfrentam lesões meniscais.

## Introduction

The meniscus consists of a fibrocartilaginous structure of significant importance in maintaining joint homeostasis, and it plays a fundamental role in several functions, including load distribution, impact absorption, joint stabilization, proprioception, and lubrication. However, a radical meniscectomy can substantially compromise these vital functions. Meniscal injuries may be traumatic or degenerative, thus covering a wide range of clinical situations. The therapeutic approach to these injuries ranges from conservative options to surgical interventions. 1

## Lesion Classification

The classification of meniscal injuries relies on their morphology, location, orientation, and extension.

Regarding location and extension, the lesions can affect the anterior and posterior horns, the body, and the anterior and posterior roots. Smigielski et al., 2 modified this topographic classification by subdividing the regions per their peculiarities.

As for orientation, meniscal injury classification is as follows: longitudinal, when affecting the circumference of the meniscus, parallel to the central fibers, or radial, that is, vertical, perpendicular to the circumference, and crossing the central fibers. A longitudinal injury can also be horizontal (separating the meniscus into upper and lower portions), oblique, or vertical. Vertical injuries can generate a large fragment that moves to the intercondyle, the so-called “bucket handle injury.” Radial lesions can be straight or curved; the latter causes the formation of flaps. Lesions with anomalous presentations, that is, in multiple directions, are deemed complex. 3

Meniscal injuries can also be classified according to magnetic resonance imaging (MRI) findings as grades I, II, and III. Grade-I lesions are globular and present an intrameniscal hypersignal that does not extend to the articular surfaces; they represent the initial degenerative changes of the meniscus. Grade-II lesions present an extensive hypersignal with no defined cleavage plane, and they may extend to the meniscal margins, but with no clear communication with the joint cavity; they represent more advanced degenerative changes. Grade-III lesions present a well-defined hypersignal and communicate with the joint surfaces, denoting a traumatic origin. 4

## Conservative Treatment

Conservative treatment is indicated in oligosymptomatic patients with stable lesions, and as the first treatment in cases of degenerative meniscal lesions. Imaging findings compatible with meniscal injury are not rare, even in asymptomatic patients, and one must be careful not to overindicate surgical treatment, 5 especially due to the lack of evidence that surgical outcomes from partial meniscectomies are superior to those of the long-term conservative treatment. Therefore, the surgical approach is more frequent in patients with mechanical symptoms and injuries with an instability pattern, such as flaps or bucket handles. 6 7 8

Conservative treatment includes physical therapy to improve pain and treatment of muscular and proprioceptive deficits in combination with a supervised exercise program focused on muscle strengthening of the hips and knees. 9 10

## Surgical Treatment

### Partial Meniscectomy

Partial meniscectomy is a surgical procedure indicated in injuries in which the repair with suture techniques is not feasible, or in injuries with low healing potential. With a better understanding of the meniscal function, the approach should be restricted to the affected area, seeking a minimal resection to obtain firm and regular residual edges. Debridement of the injured meniscal portions employs manual instruments like arthroscopic scissors of different orientations or motorized equipment, such as a shaver.

The postoperative period is usually not very demanding, with weight-bearing and mobility allowed early. Although partial meniscectomy is associated with short-term improvement in symptoms, it generates biomechanical changes in the knee and an increased risk of joint degeneration compared with suture techniques, and it may be associated with acute loss of impact absorption and alterations in subchondral bone homeostasis, to the point that it may progress to insufficiency fracture or postarthroscopy osteonecrosis.

## Meniscorrhaphy

### Outside-in Suture

Outside-in suturing techniques enable repairs using simple and widely-available materials, reducing technical costs. They are particularly useful for injuries in most anterior meniscal regions and have limitations in cases of more posterior injuries. They may be performed by passing needles directed towards the area to be repaired from the outside to the inside, thus taking suture threads that may be retrieved through arthroscopic portals, enabling the transport of one of the threads to the desired location. 11 12 An alternative technique involves using a needle with a suture thread folded as a loop carrying another thread for passage through a second needle introduced with the same technique. Needle manipulation enables passing between the suture threads with no need for their retrieval through the portals. 13

After suturing as desired, a small access can be made at the site for suture tying. During this step, one must check for the inexistence of anatomical structures, such as the saphenous nerve, which should not be tied between the ends of the suture. Alternatively, suture thread retrieval can occur through the portals after subcutaneous dissection with an arthroscopic grasper, which enables the knot to be tied and directed using arthroscopic knot pushers. 14

### Inside-out Suture

In this technique, the meniscal suture needles are directed through cannulas from inside the joint to the external region and retrieved through accesses made in the medial or lateral region, thus transporting the threads for suturing. Its cost is higher because it requires suture needles and surgical threads. However, the same needle kit may be used to perform several sutures, providing greater versatility in the direction and number of stitches. It is considered the gold standard technique. 11 15

Its disadvantage is the need to perform surgical accesses to protect noble structures that may undergo perforation or be inadvertently trapped between the knots. In the medial region, an access is recommended to protect the saphenous nerve, and, in the lateral region, likewise, the access is recommended with the goal of protecting the peroneal nerve. The location of the medial access is planned posteriorly to the medial collateral ligament, with approximately 3 cm to 6 cm, with one-third of the ligament proximal to the joint line and two-thirds distal to it, and deep dissection in the plane between the medial gastrocnemius and the posterior edge of the tibia must be performed. Instruments similar to a spoon may be used to move away and direct the needles posteriorly during the progression from the joint. 15

In the lateral region, the indicated access is performed with approximately 3 cm to 6 cm, and its center is in the joint interline posterior to the lateral collateral ligament. between the biceps femoris and the iliotibial tract. A dissection of the deep planes is performed in the interval between the lateral gastrocnemius and the joint capsule, sparing neurovascular structures in the region during needle targeting and retrieval.

The inside-out technique enables the adequate repair of injuries to the body and posterior meniscal region. Nevertheless, it has disadvantages, including the need to perform an access and to have a qualified assistant perform the technique step by step, as well as an increased operative time compared with all-inside techniques.

### All-inside Suture

All-inside suture devices are specific materials for meniscal sutures that enable the insertion of stitches across the joint without the need to create an access to retrieve the suture threads. The first generation of the meniscal suture technique used curved suture hooks through posterior arthroscopic portals to make stitches through the tying technique with arthroscopic knots. 16

With the evolution in the materials available, a second generation of the technique enabled the use of specific devices to anchor the threads in the capsule by means of an access through the conventional portal. As in the first generation, it requires the performance of knots by the surgeon using an arthroscopic technique. 17

The third generation of devices used wires coupled to rigid darts anchored in the joint capsule, enabling suture tension for the performance of the planned repair. As a disadvantage, these devices resulted in chondral damage due to the size and rigidity of the darts. 18 19

A fourth generation of devices was developed, which overcame the disadvantages of third-generation devices and improved technical outcomes. These devices are specific materials, with threads coupled to low-profile materials used for peripheral anchoring of the sutures and loaded knots enabling tensioning and tightening the suture as needed. These devices yield outcomes equivalent to those achieved with the inside-out techniques, with greater comfort for the surgeon, and reduced operative time. However, their cost is higher, which limits their use. 11 20

Recently, new devices have been developed, which enable pin modeling to release the anchoring systems coupled with wires, reaching lesions in regions of difficult access. In addition to these devices, specific materials to perform all-inside sutures with transfixation of the meniscal tissue without the need for peripheral capsular anchorage were created, enabling the execution of circumferential repairs in different configurations according to the surgical planning. These instruments also facilitate the execution of transosseous meniscal reinsertion with pull-out techniques to treat meniscal root injuries.

## Special Situations

### Treatment of Radial Lesions

Radial meniscal injury consists of a vertical injury to the meniscus, perpendicular to its circumferential axis, starting from the free edge of the meniscus and running to its peripheral portion. This injury can be partial, not reaching the meniscocapsular junction, or complete, involving the entire structure of the meniscus and separating it into two fragments (anterior and posterior to the injury). 21

The treatment of radial meniscus injuries has evolved with a better understanding of meniscal function and technical advances in surgery. In the first descriptions of this injury pattern, in 2004, Bin et al., 22 reported that the repair was virtually unfeasible because of the meniscal quality and low healing potential. However, suturing these injuries yielded good outcomes with symptomatic improvement and functional restoration. 23

Meniscectomy has been the standard treatment for this type of injury for a long time. 24 In partial injuries affecting the central and avascular portion of the meniscus, debridement until obtaining a stable edge with rounded contours is a proper treatment, as it preserves as much of the native meniscus as possible and reduces the risk of peripheral progression of the injury. However, evidence of changes in load biomechanics and pressure distribution even in small meniscal resections, as well as recent advances in meniscal repair techniques, resulted in an increased trend to suture these injuries. 25

Radial injury repair aims to reestablish the ability of axial stress absorption, load transmission, and meniscal extrusion prevention. The suture methods for meniscal injuries have evolved significantly, with different devices to performing inside-out, outside-in, and all-inside sutures. It is critical to master these techniques when faced with complex meniscal and radial injuries, as technical combinations are often required for proper treatment.

The most traditional suture pattern is horizontal, perpendicularly crossing the lesion plane with a 5-mm distance between the most peripheral and the central points and meniscal anchorage 5 mm from the lesion. 26 In 2012, Matsubara et al. 27 modified the technique, showing that crossed (or “X”) sutures are mechanically superior to double parallel sutures, with higher maximum tension and rigidity. The oblique suture orientation concerning the circumferential collagen fibers may provide the mechanical advantage of this suture pattern.

Another technical modification was described by Bhatia et al., 28 who associated transosseous anchorage with the traditional horizontal suture to improve the mechanical resistance of the repair, control extrusion, and biologically stimulate the repair with factors originating from bone tunnels. In this technique, each free edge created by the radial injury is looped in its most peripheral portion; next, these two sutures are brought through two transosseous tunnels and sutured over a button attached to the anterior cortex of the tibia. Adding the transosseous suture to the radial injury suture promoted a two-fold increase in the maximum tensile strength compared with the isolated suture, as well as a significantly smaller diastasis between the meniscal stumps. This technique seems especially valid for the medial meniscus because of its less mobile nature. However, it requires further studies on the potential anchoring effects on the lateral meniscus.

New all-inside suturing instruments have been released recently, and they enable circumferential meniscal suturing. These circumferential sutures showed smaller meniscal openings after testing with 100, 300, and 500 cycles, greater stiffness, and greater strength to failure. 20

## Treatment of Meniscus Root Lesions

Root injuries are those that occur up to 10 mm from the meniscal tibial insertion. They are relevant because of their biomechanical behavior, which is equivalent to a complete meniscectomy due to the loss of meniscal circumferential tension and consequent meniscal extrusion. The associated effects of increased peak pressure and reduced contact area result in a natural history of rapid progression of the degenerative process and collapse of the affected compartment. 29 30

The injuries can be traumatic or degenerative. Traumatic injuries frequently affect the posterior root of the lateral meniscus in young patients, and they are associated with an acute sprain of the ligament injury, especially the anterior cruciate ligament (ACL). Degenerative lesions of the posterior root of the medial meniscus present in five patterns. 31

**Type 1–** Partial stable lesion within a radius of 9 mm from the center of the root attachment;

**Type 2–** Complete radial lesion within a radius of 9 mm from the center of the root attachment;

**Type 3–** Bucket handle with a full offset within a radius of 9 mm from the root attachment;

**Type 4–** Complex oblique pattern within a radius of 9 mm from the root attachment; and

**Type 5–** Avulsion fracture of the meniscal root from the tibial plateau.

Degenerative lesions are most frequent in women in the fifth and sixth decades of life. These lesions classically cause clicking in the posterior region of the knee after activities involving excessive knee flexion or descending stairs. The knee begins to experience moderate joint effusion, which can lead to significant pain when walking, in addition to pain on the inner side of the knee due to injury to the posterior root of the medial meniscus. 31

**Fig. 1:**
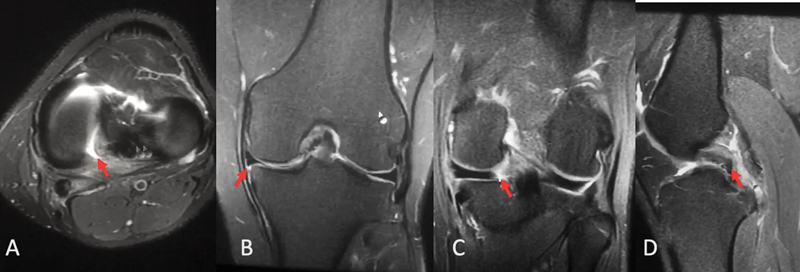
Magnetic resonance imaging scan of a patient with posterior meniscal root injury. ( **A** ) Axial acquisition. ( **B** ) Coronal acquisition, indicating extrusion. ( **C** ) Lesion identified in the coronal acquisition. ( **D** ) Sagittal image.

The diagnosis consists of clinical history and specific signs on the MRI examination ( Fig. 1 ). This injury identification relies on the truncation sign (loss of continuity of the posterior portion of the meniscus in the coronal section), the phantom meniscus sign (absence of the meniscal image in the sagittal section), or a complete radial lesion close to the root. Meniscal extrusion, that is, a distance greater than 3 mm between the capsular meniscus border and the tibial plateau limit, demonstrates the loss of meniscal circumferential containment, and is an indirect sign of root injury. 32 Routine radiographs of the knee under load and panoramic radiographs to assess the axis of the lower limb are part of the diagnostic investigation.

Treatment relies on symptomatic relief, joint biomechanics restoration, and osteoarthritis prevention. The treatment of meniscal root injuries can be conservative or surgical. Conservative treatment is reserved for elderly, sedentary patients who already present subchondral bone collapse or advanced osteoarthritis. In patients with advanced meniscal extrusion, poor meniscal quality, and obese subjects, surgery is an exceptional procedure. Non-surgical treatment yields poor outcomes, with a natural history of progressive evolution of the degenerative process, with rates of progression to arthroplasty ranging from 30 to 45% in 5 years. 33

The ideal candidates for surgical treatment are patients with traumatic injuries associated with ligamentous or acute degenerative injuries, little extrusion, and Outerbridge 1 or 2 chondral injuries. Patients with limb misalignment must undergo alignment surgery simultaneously or before meniscal injury treatment.

The surgical treatment reportedly recovers the biomechanics of native load distribution of the meniscus after repair, improves functional scores, and results in lower rates of conversion to arthroplasty. 34 35

Suturing meniscal root injuries is performed through a specific technique that requires special devices to transfix the menisci (all-inside forceps or hook-type suture passers), passing ultra-resistant threads through the meniscal root stump. Then, specific guides create bone tunnels in the tibial root to transport the wires, fixating the meniscal stump in the tibial bone, and creating a new meniscal root. These sutures reach the anterior cortex, fixating the menisci. The postoperative period is highly restricted, with load and excessive knee flexion limitations for more than 6 weeks, followed by progressive loading and strengthening. 36

## Treatment of Degenerative Meniscal Lesions

Degenerative meniscal injury is characterized by progressive changes in the structure and function of the knee menisci, which result in deterioration over time. Meniscal degeneration is often associated with the natural aging process, which decreases meniscal elasticity and strength. This gradual degeneration makes the menisci more susceptible to injury, even during low-impact everyday activities. Repetitive movements, excessive twisting, or continuous overload can result in tears or other injuries to already degenerated menisci. 37

The diagnosis of degenerative meniscal injury typically consists of clinical evaluation and imaging tests, such as MRI, which provides a detailed visualization of the knee structures and helps determine the extent of the injury. 38

Treatment must be tailored to the patient depending on symptom severity, age, physical activity level, and overall joint degenerative status. Radiographic evaluation is essential to understand the injury as an isolated source of pain or as part of a more diffuse joint degeneration process. Moreover, it evaluates the limb axis, as osteotomy can be a critical strategy for pain relief and joint longevity increase. 39 40

Treatment strategies include rest, cryotherapy, physical therapy for muscle strengthening, and non-steroidal anti-inflammatory drugs for pain and inflammation control. If these treatments are unsuccessful, corticosteroid infiltrations or viscosupplementation are frequent strategies.

The surgical treatment of degenerative lesions is exceptional, as several clinical trials failed to show better outcomes after it. 9 10 39 40 41 Therefore, surgery is best indicated in cases of failure of the conservative treatment or the presence of mechanical symptoms in the joint, such as joint locking and blocks, and it must be performed with resection of the smallest meniscal mass possible to avoid complications such as progression to osteoarthritis and postarthroscopy osteonecrosis. 37

The surgical approach to degenerative lesions with a horizontal pattern involves special challenges. The greater distance between the edges of the lesion and the higher degree of associated cartilaginous damage negatively affects the surgical outcomes. In addition, the presence of mechanical symptoms is not a predictive factor of surgical success. 42 In meniscectomy, complete resection of the lower leaflet of the lesion appears to present inferior clinical outcomes than those of partial resection. 43 It is also possible to perform circumferential suture techniques in the meniscus. Although associated with low healing rates, these techniques yield good clinical outcomes. 44

## Treatment of Meniscal Ramp Injuries

Meniscal ramp injuries occur in the peripheral region of the posterior horn of the medial meniscus, and their diagnosis requires a high index of suspicion. Imaging exams, such as the MRI, may not present the specific signs of injuries shown by arthroscopic exploration, so a careful routine of evaluation must be performed. 45 46 47

The risk factors for this type of injury appear to be the male gender, age under 30, posteromedial tibial edema on MRI, concomitant lateral meniscal injuries, complete ACL injuries, and chronic ligament injuries. 48 Some studies 49 50 51 suggest that stable meniscal ramp injuries do not require suturing, allowing for an approach with debridement and ligament stabilization alone. Injuries larger than 2 cm with detected instability require meniscal suture repair to avoid persistence of the instability or reconstruction failure. 47 49 51

Arthroscopic evaluation begins with an assessment of the mobility of the medial meniscus with palpation using a probe. However, conventional arthroscopic portals are often insufficient for adequate injury diagnosis, requiring the adoption of posterior visualization through the space between the posterior cruciate ligament and the medial femoral condyle. A posterior arthroscopic portal may be performed to palpate the meniscocapsular transition and used for the indicated suturing techniques. 47 52

Ramp injuries are classified according to the following five types: 53

**Type 1–** Meniscocapsular injuries, which are very peripheral. Mobility during anterior palpation with the probe is low;

**Type 2–** Upper partial lesions, which are stable, and their diagnosis relies on the posterior view alone. They also present low mobility during probe testing;

**Type 3–** Inferior or hidden partial lesions, which, at first, are not visible in the posterior view, but one can strongly suspect them when there is significant mobility with probe testing;

**Type 4–** Complete rupture in the red-red zone. Mobility is very high during probing;

**Type 5–** Double injury.

**Fig. 2:**
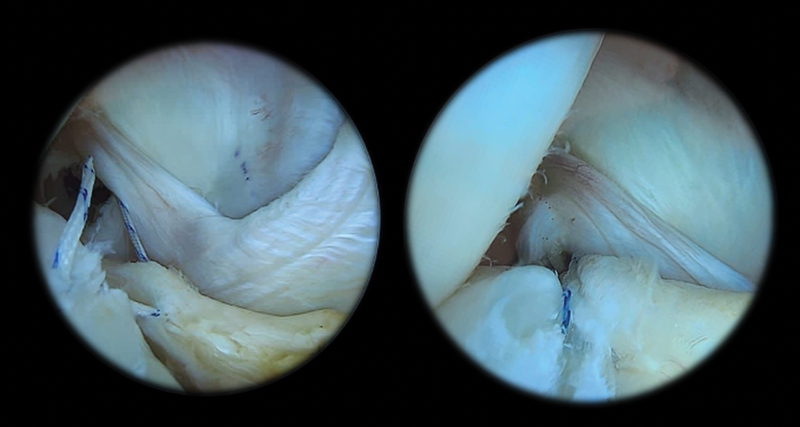
Visualization through the intercondyle of a meniscal ramp injury with suture through the posterior portal.

Different repair techniques may be applied, including the approach through a posteromedial portal using suture hooks ( Fig. 2 ) or transfixing forceps. 47 54 Suturing using all-inside devices is an option, though there is a concern regarding potential failure due to the lack of anchoring of the devices in the capsule and the possible need for technical modifications for proper repair using these devices. 55 56 57 58

Although the number of studies investigating the treatment of meniscal ramp injuries has increased in the last few years, there are few studies with a high level of evidence available, and further research is required to clarify some questions on the matter.

## Meniscal Transplantation

Meniscal transplantation is a rescue alternative for symptomatic relief, functional improvement, and to delay the degenerative process of the joint in patients undergoing previous total or subtotal meniscectomy. However, patient selection requires care.

The presence of inflammatory arthritis, immunodeficiency, previous infection, and severe osteoarthritis contraindicate the procedure. Although they are not absolute contraindications, condylar flattening or the presence of osteophytes are associated with a worse prognosis. Other conditions, such as obesity, ligament instability, axis deviation, and focal cartilage defects, must be corrected before or during transplant surgery. 59 60

A physical examination of previous scars, range of motion, ligament instability, poor alignment, pain during joint line palpation, and a complete radiological evaluation must complete the clinical history.

**Fig. 3:**
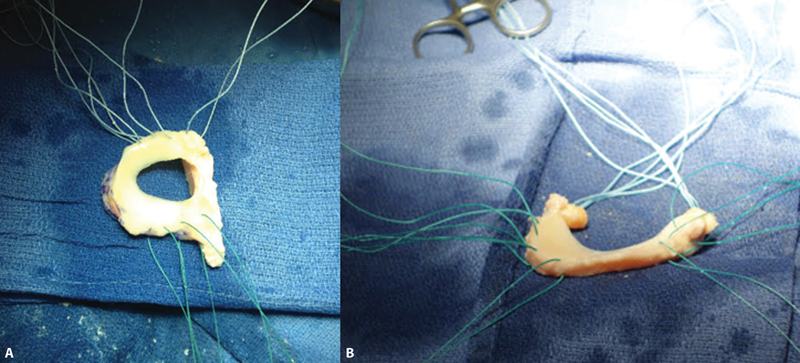
( **A** ) Lateral meniscus allograft with bone crest. ( **B** ) Medial meniscus allograft with bone plugs.

The transplant surgical technique can be open or arthroscopic, which presents lower morbidity. The size of the meniscus is estimated using anteroposterior and lateral radiographs. The most commonly used fixation methods include a bone plug under the meniscal roots or a bone crest ( Fig. 3 ). After bone fixation, the meniscus should be sutured close to the joint capsule using one of the several meniscal suture techniques (all-inside, inside-out, and outside-in). 61

## Rehabilitation

The postoperative period of arthroscopic meniscectomy involves the early release of complete weight-bearing and the use of crutches may be adopted for 1 to 2 weeks according to pain. Braces or immobilizers are not required, and the recommendation is for the early start of a program to gain range of motion, beginning with muscular and proprioceptive deficit recovery to enable the return to training and sports practice. 62

The postoperative period for meniscal sutures depends on the injury pattern and the suturing technique used. In longitudinal injuries, axial load can be allowed using knee immobilizers or braces. However, in radial injuries, the patient is advised not to place any load on the operated limb to minimize the risk of meniscal diastasis or suture rupture for 4 to 6 weeks. The range of motion up to 90° is encouraged in the first 3 to 4 weeks to avoid joint stiffness; next, it is allowed sequentially until completion, and the single limitation is to perform deep flexion in a closed kinetic chain for 4 to 6 months. 62

## Final Considerations

The treatment of meniscal injuries has evolved by recognizing previously undiagnosed injury patterns and developing materials and techniques to facilitate meniscal sutures. The conservative treatment still seems to be a good strategy for patients with degenerative lesions. Avoid meniscectomy as an initial strategy, except when suturing techniques are not feasible or when they may increase the risk of joint degeneration in the long term. Specific injuries represent a separate challenge that will lead to the future development of new treatment strategies.
